# Cu(i) substituted wurtzite ZnO: a novel room temperature lead free ferroelectric and high-*κ* giant dielectric†

**DOI:** 10.1039/d0ra00933d

**Published:** 2020-03-20

**Authors:** Neeraj Singh, Preetam Singh

**Affiliations:** Department of Ceramic Engineering, Indian Institute of Technology (Banaras Hindu University) Varanasi Uttar Pradesh India 221005 preetamsingh.cer@itbhu.ac.in preetamchem@gmail.com +91-9473720659

## Abstract

Semiconducting wurtzite ZnO, with the highest incipient piezoelectricity is an attractive alternative choice with doping transition metal ions in the host lattice to develop novel binary ferroelectric materials that can be easily fabricated in any device architecture. Up to 8% Cu^+^ ion substitution on Zn^2+^ sites in the ZnO lattice was achieved by careful selection of raw material and adaptation of a low temperature sol–gel synthesis route for the preparation of bulk material. Phase purity and substitution of Cu^+^ ions in the ZnO lattice along with oxide-ion vacancy formation was confirmed using Powder X-ray diffraction (XRD), Scanning Electron Microscopy (SEM), and Energy Dispersive X-ray analysis (EDX), X-ray Photoelectron Spectroscopy (XPS) and Magnetic property measurement system (MPMS) studies. A giant dielectric constant (∼6300) was observed at 600 °C for Zn_0.95_Cu_0.05_O_1−*δ*_ pellets at 100 kHz frequency. Bulk Zn_0.95_Cu_0.05_O_1−*δ*_ also exhibits ferroelectricity at room temperature with remnant polarization *P*_r_ and *V*_c_ equal to 9.60 × 10^−3^ μC cm^−2^ and 3.83 × 10^2^ V cm^−1^ respectively.

## Abstract 2

Cu^+^ ion substituted ZnO, Zn_1−*x*_Cu*_x_*O_1−*δ*_ have shown high dielectric constant (∼6300) at 600 °C at 100 kHz frequency and ferroelectricity at room temperature than for bulk Zn_0.95_Cu_0.05_O_1−*δ*_ samples.

## Graphical abstract

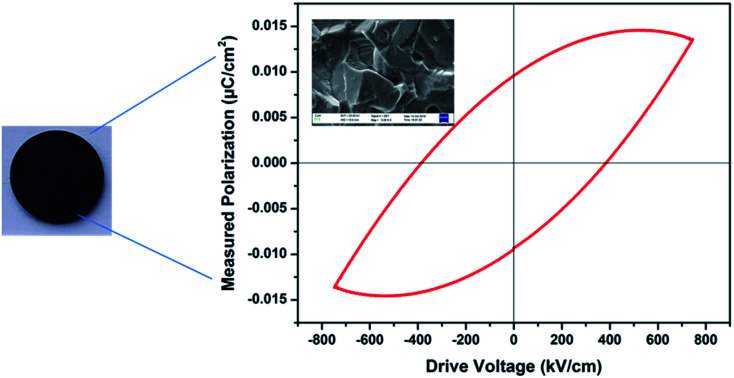

## Introduction

Semiconducting ferroelectric materials have technological importance due to extensive applications in optoelectronic devices for uses in optical communication, memory, displays and coherent optical processing.^1–6^ The specific device applications also include capacitors, modulators, beam deflectors, light valves and holographic storage media. Further, one of the efficient ways to scavenge electrical energy through vibration based energy harvesting is by utilization of piezoelectric and ferroelectric materials. Ferroelectric bulk crystals are desired for usage in optoelectronic devices because of the combination of extraordinary optical and electronic properties originated from their non-centro symmetric crystal structure and structural anisotropy that exhibit many nonlinear optical properties. Using field effects coupled with high-*κ* dielectrics and ferroelectric polarizations, the electric properties of ferroelectric semiconductors have been widely tuned to facilitate the emergence of various gate-modulated devices such as transistors, logic inverters and memory storage devices, light-emitting diodes (LEDs) and photo detectors.

In semiconducting materials, ferroelectricity appears mainly by the delicate balance between a long-range dipole–dipole interaction and a short-range interaction. Slight distortion of electron cloud due to structural changes in dielectrics gives a rise of dipole moments. The ferroelectric phase transitions are associated with a structural phase transition from high symmetry paraelectric phase to the low symmetry ferroelectric phase. Therefore, new materials with a simple structure are not only preferable for understanding the microscopic origin of ferroelectricity, but are also important for integrating into modern ferroelectric devices with utilization of novel ferroelectric/piezoelectric/relaxor and multiferroic materials that do not contain environmental hazards such as Pb and Bi. Also, there is an interest to bring d^0^ and d^*n*^ (0 ≤ *n* ≤ 10) transition metal atoms together in a simple oxide matrix to realize multiferroic properties. Wurtzite piezoelectric ZnO, with highest piezoelectricity can be attractive alternative choice with doping transition metal ion in host lattice to develop novel ferroelectric materials.^7–9^ Zinc oxide (ZnO) is considered as one of the very important metal oxides due to its unique chemical and physical properties such as electrochemical coupling, good chemical and photo stability and wide range of radiation absorption capabilities.^10,11^ Further ZnO is a very low-toxicity semiconductor that have been implemented successfully in biomedicine and in pro-ecological systems.^12,13^ Although pure ZnO has the possibility to exhibit ferroelectricity, the polarization switching does not observed until its melting point (1975 °C) because of large activation energy accompanied by dipole switching process.

ZnO has also been considered for spintronic applications: if doped with 1–10% of magnetic ions (Cu, Mn, Fe, Co, V, *etc.*), ZnO could become ferromagnetic even at room temperature.^14–17^ Substitution of ZnO with different elements such as Fe, Al, Ni, Cu and Mg, can optimize the band gap of ZnO and that will give the needed flexibility for application in modern opto-electronic devices.^18–20^ Cu is one of promising dopants because of its high abundance and low toxicity and multiple electronic state (Cu^+^/Cu^2+/^Cu^3+^) formation that can have inbuilt effect of structural phase transitions. Therefore, the study was aimed to investigate the interaction of d^10^ cations (Zn^2+^ and Cu^+^) in tetrahedral symmetry and role of resultant oxygen vacancy generated through incorporation of Cu^+^ ions in host ZnO lattice on dielectric interaction/polarizations and search of a possible ferroelectric or ultra-high *κ* dielectric material. There is some report on ferroelectricity in Cu doped ZnO thin films that may be arises due to formation surface oxygen vacancy in ZnO thin films fabricated ultra-vacuum conditions.^21,22^ However, there is no report on appearance of ferroelectric phase in bulk ZnO based materials. Thus, it was important to synthesize Cu^+^ doped ZnO in bulk and study dielectric properties and search the ferroelectric transition in bulk material. For the first time by employing selective precursor, Cu(i)Cl and sol–gel method, we have able to synthesize Cu(i) doped ZnO that show high-*κ* dielectric and ferroelectricity in bulk. Synthesis, characterization and study of dielectric/ferroelectric properties of Cu(i) doped ZnO microcrystals are presented in this manuscript.

## Experimental section

### Synthesis and characterizations

Cu doped ZnO; (Zn_1−*x*_Cu_*x*_O_1−*δ*_) ceramic sample were prepared by the modified sol–gel route in the range of *x* = 0.02 to 0.15. In the molar ratio, (1−*x*) : *x* : 1.5 ZnO, CuCl and EDTA were taken for sample preparation. For Zn_0.95_Cu_0.05_O_1−*δ*_, 95 mmol ZnO is dissolved in dilute nitric acid on hot plat at 200 °C with continuous stirring and after obtaining clear solution, 5 mmol CuCl was added into it, the colour of solution turned into greenish blue. After this, ammonia was added drop by drop till sky blue colour was appeared. Further, 150 mmol EDTA (ethylene diamine tetra acetate) was added for chelation.

**Fig. 1:**
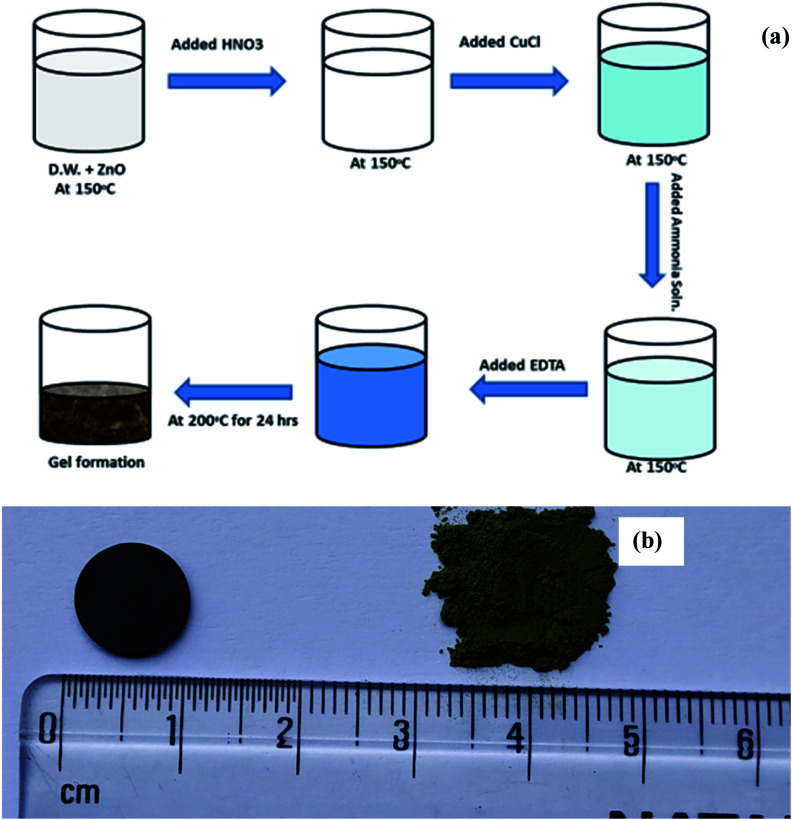
(a) Schematic of the synthesis scheme of Cu doped ZnO; Zn_1−*x*_Cu_*x*_O_1−*δ*_ and (b) photograph of synthesized powder and sintered pellet of Zn_0.95_Cu_0.05_O_1−*δ*_.

The solution is now heated at 200 °C with continuous stirring till dryness and kept in a hot air oven at 200 °C for 10 h to remove moisture. The powder was ground after that in mortar pestle and kept for calcinations in alumina crucible at 800 °C for 15 hours. Further the material was reground and further heated at 1050 °C for 12 h. The resultant powder obtained was of green colour. The schematic of synthesis procedure is presented in Fig. 1(a) of pellets were made by hydraulic press at 8 tonne in a die of ∼1 cm thickness and the green pellet was heated at 1050 °C for 15 h twice to obtain a dense pellet. Images of prepared Cu substituted ZnO powder and obtained pellet was also shown in Fig. 1(b). The density of the pellet was measured using Archimedes method was 97% of the theoretical density.

The structure and phase purity of Cu doped ZnO samples were analysed by powder X-ray diffraction (XRD) analysis. XRD was carried out using RIGAKU diffractometer (Smart Lab 9 kW, target: Cu Kα, *λ* = 1.5408 Å). Structure of the samples were refined using Rietveld method taking hexagonal *P*6_3_/*mmc* wurtzite ZnO as model structure. Microstructure of the powder as well as pellets were analysed by scanning electron microscopy. The EDX analysis was carried out by the probe connected to SEM instrument to confirm the composition of the samples. Scanning electron microscope (SEM) and Energy-dispersive X-ray spectroscopy (EDX) studied were carried out by EVO-18 Research, ZEISS. XPS studies were carried out to evaluated electronics structure of the materials. X-ray photoelectron spectra (XPS) of the sample were recorded in a Thermo Scientific Multilab 2000 instrument using Al Kα operated at 150 W. Binding energies reported here are with reference to C (1s) at 284.5 eV and they are accurate within 0.1 eV. The complex impedance spectroscopic study of Cu doped ZnO pellets were made by Metrohm Autolab (PGSTAT204) equipped with FRA32M module. Impedance measurements were analysed using NOVA software. The two-probe ac impedance measurements were made in air in the frequency range from 1 Hz to 1 MHz with applied ac amplitude of 20 mV. Two Ag blocking electrodes were made by coating Ag paste (from Heraeus) on the two faces of the pellets and baking at 400 °C for 1 h.

For the ferroelectric studies, polishing of the pellets were carried out by fine grain size emery paper followed by heat treatment at 600 °C to remove residual stresses from the pellet. After that electroding was done on the both side of pellet by silver paste. Further pellet was heated at 400 °C for 1 hour to dry the electrodes fabricated on the pellet. Ferroelectric measurement was carried out at room temperature from 100 Hz to 500 Hz frequency range using RADIANT precision premier II. For magnetic studies of our sample Zn_0.95_Cu_0.05_O_1−*δ*,_ fine ground powder was subjected to magnetic property measurement system (MPMS) by Squid based magnetometer (MPMS-3, Quantum Design Inc.).

## Result and discussion

**Fig. 2:**
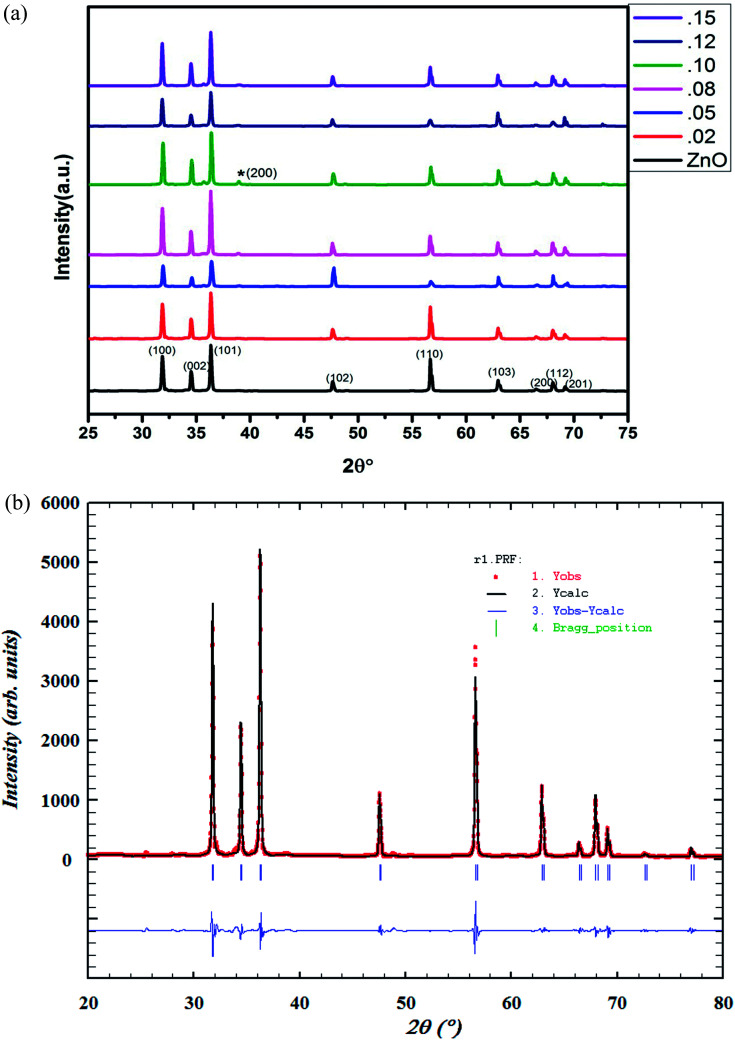
(a) Powder XRD pattern of Zn_1−*x*_Cu_*x*_O_1−*δ*_ (*x* ≤ 0.15) samples. (b) Rietveld refined powder XRD pattern of Zn_0.95_Cu_0.05_O_1−*δ*_.

The structural parameters and phase purity have been studied using the powder X-ray diffraction patterns. XRD patterns of Zn_1−*x*_Cu_*x*_O_1−*δ*_ (*x* ≤ 0.15) is shown Fig. 2(a). Sharp and intense diffraction peaks of all the samples confirm high crystallinity of samples. It also reveals a clean hexagonal phase formation (sp. gp *P*6_3_*mc*) in wurtzite ZnO structure for full doping range along with minute impurities of CuO phase at higher Cu doping concentrations.

Cu^+^ is not stable in air. It radially oxidize to Cu^2+^, that is why Cu^+^ ion impurities that is not substituted in ZnO lattice appears as CuO (Cu^2+^ oxide). Cu^+^ ion is stable in air only in the form of CuX (X = Cl, Br and I). For the preparation of Zn_1−*x*_Cu_*x*_O_1−*δ*_, we have heated our sample in air that is why CuO was formed as impurity phase.

Using FullProf software, structure of materials was refined by Rietveld method. The Rietveld refined XRD image of Zn_0.95_Cu_0.05_O_1−*δ*_ phase is shown in Fig. 2(b). The absence or negligible amount of diffraction peaks for metallic Cu, Cu_2_O and CuO phases suggest the substitution of Cu in ZnO lattice without altering its wurtzite structure. Structural parameter derived from Rietveld refinement of Zn_1−*x*_Cu_*x*_O_1−*δ*_ (*x* ≤ 0.12) is given in ESI (Table S1†). The change in lattice parameter of Zn_1−*x*_Cu_*x*_O_1−*δ*_ is almost negligible. This may be due to the fact that in tetrahedral coordination, ionic radius of Cu^+^ is 0.74 Å and Cu^2+^ is 0.71 Å and that is almost same or close to the ionic radii of Zn^2+^ (0.74 Å) in tetrahedral coordination.^23^ That is why, during lattice formation, Cu ions easily able to occupy on Zn sites in wurtzite crystal lattice. However, with the increase in doping percentage of Cu, (*x* ≥ 0.08), weak diffraction peaks corresponding to CuO phase start appearing in the powder XRD pattern (Fig. 2(a)) due to segregation of cu ions. Thus, only up to 8% Cu ions can be substituted on Zn^2+^ sites in ZnO lattice.

SEM images of (a) powder and (b) intersection of pellet of Zn_0.95_Cu_0.05_O_1−*δ*_ in ESI Fig. S1.† The SEM study reveals that the grains are of micrometre sizes it the range of 2–50 μm and hexagonal shape. The pellets are well-sintered, and the grains were in good contact with each other. The EDX image shown in ESI Fig. S1(c)† also confirms the composition and apparent homogeneity of the materials. EDX study has been carried out by the EDX probe attached in the SEM instrument (Fig. S1†) and indeed Zn and Cu were found in the ratio of 0.95 : 0.05, which agrees well with the composition taken for the preparation. The density of the pellets was measured by the Archimedes method in water and was ∼97% of the theoretical density of the material.

**Fig. 3:**
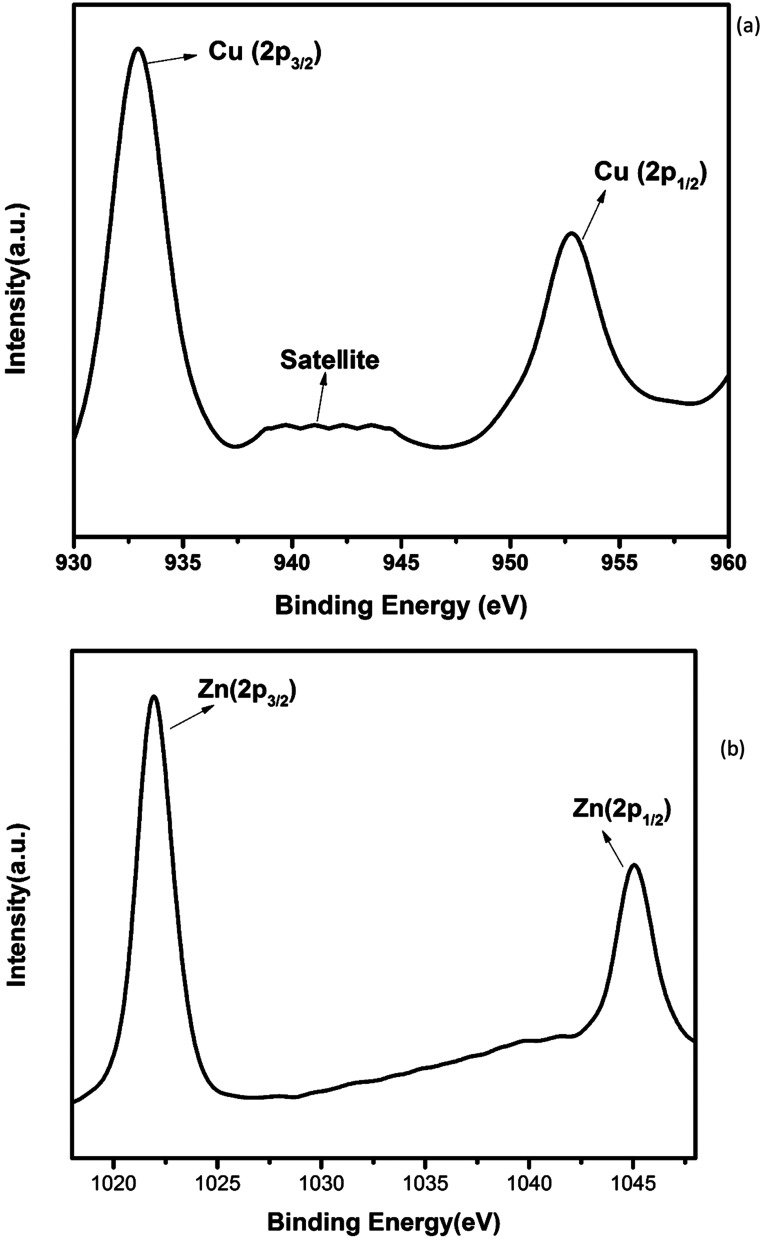
Core level XPS spectra of Zn_0.95_Cu_0.05_O_1−*δ*_. (a) Cu (2p) and (b) Zn (2p).

The electronic structure and oxidation states of Cu and Zn in Zn_0.95_Cu_0.05_O_1−*δ*_ samples were investigated by X-ray photo-electron spectroscopy. Core level Cu (2p) spectra of Zn_0.95_Cu_0.05_O_1−*δ*_ sample is shown in Fig. 3(a). Binding energy of Cu (2p_3/2_) is observed around 932.9 eV and a very broad and weak satellite peak was observed at 10 eV from the main peak confirms that Cu ions are mostly is in +1 oxidation state. In general, both Cu and Cu^+^ (2p_3/2_) electron have binding energy close to 933 eV with absence or very weak presence of satellite peak.^24,25^ For Cu^2+^ ions, the binding energy of 2p_3/2_ core electrons are 933.5 eV with appearance of very strong satellite peak around 940 eV.^25^ Thus it is clear for the spectra of Cu (2p) core level shown in Fig. 3(a) that Cu is exist mostly in Cu^+^ state in Zn_0.95_Cu_0.05_O_1−*δ*_. The Zn (2p) core level spectra is shown in Fig. 3(b). Binding energy of Zn (2p_3/2_) electrons was observed at 1021.5 eV. Binding energy for 2p_3/2_ electrons for Zn (metal) and Zn^2+^ ions are 1021 eV and 1021.4 eV respectively.^25,26^ Thus, Zn is in 2+ oxidation state in Zn_0.95_Cu_0.05_O_1−*δ*_ samples. Therefore presence of Cu^+^ on Zn^2+^will generate oxygen ion vacancy Zn_0.95_Cu_0.05_O_1−*δ*_ lattice. Using Kröger Vink notation, the oxygen vacancy formation is represented as1
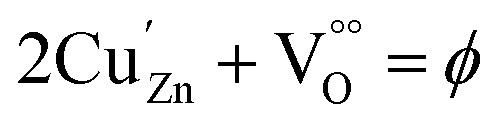
and the oxide-ion vacancy formation after Cu^+^ substitution on Zn^2+^ sites in wurtzite lattice is represented below.
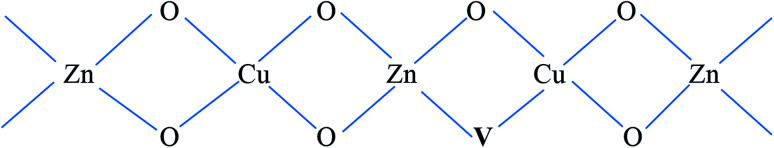


An estimate of relative concentrations of Zn and Cu were carried out from the intensities of Zn (2p) and Cu (2p) peaks in Zn_0.95_Cu_0.05_O_1−*δ*._

Relative surface concentration is calculated from the formula:^27^

2 Relative concentration *C*_M_ = (*I*_M_/*λ*_M_*σ*_M_*D*_M_)/∑(*I*_M_/*λ*_M_*σ*_M_*D*_M_)

where *I*_M_ is the integrated intensity of the core levels (M = Zn (2p) and Cu (2p)), *λ*_M_ is the mean escape depth of the respective photoelectrons, *σ*_M_ is the photoionization cross section, and *D*_M_ is the geometric factor. The photoionization cross-section values were taken from Scofield's data^28^ and mean escape depths were taken from Penn's data.^29^ The geometric factor was taken as 1, because the maximum intensity in this spectrometer is obtained at 90°. Surface concentrations of Zn and Cu are found in the ratio of 0.95 : 0.05 in Zn_0.95_Cu_0.05_O_1−*δ*_. Thus surface compositions of Zn_0.95_Cu_0.05_O_1−*δ*_ almost same as the bulk composition.

**Fig. 4:**
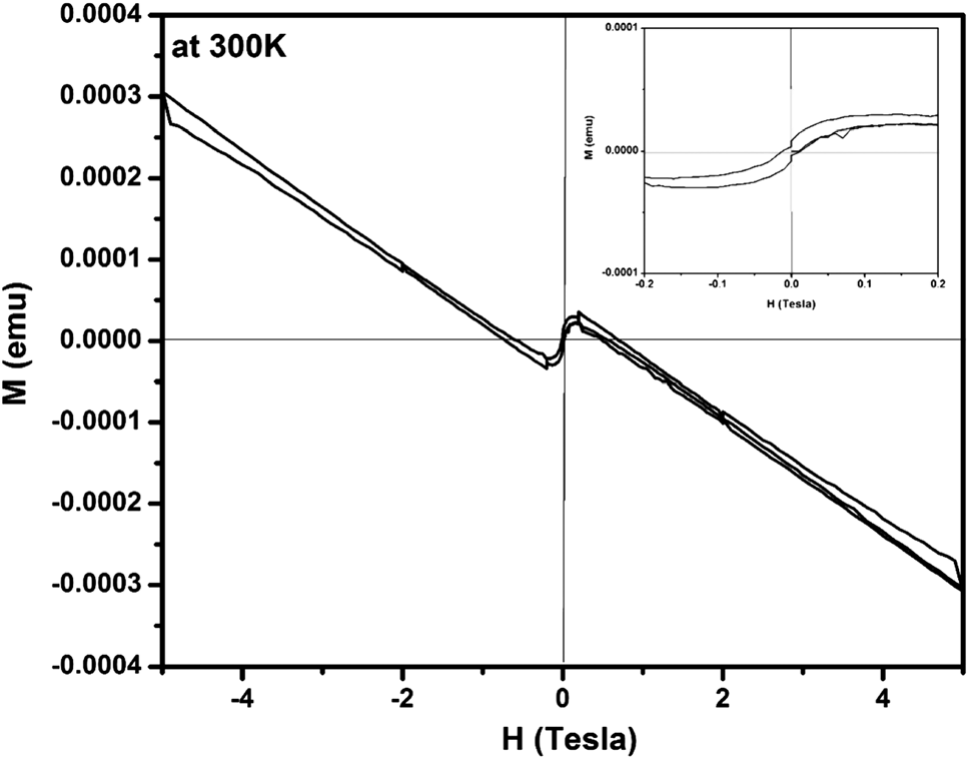
Magnetization *vs.* magnetic field (*M*–*H*) curve for Zn_0.95_Cu_0.05_O_1−*δ*_ sample at 300 K. Inset show the ferromagnetic contribution within ±0.2 T.

Further to understand the electronic structure of Zn_0.95_Cu_0.05_O_1−*δ*_ samples, magnetic response of the Zn_0.95_Cu_0.05_O_1−*δ*_ was also studied using magnetic property measurement system (MPMS) by Squid based magnetometer (MPMS-3, Quantum Design Inc.). Fig. 4 shows a typical magnetization *vs.* magnetic field (*M*–*H*) curve obtained from a Zn_0.95_Cu_0.05_O_1−*δ*_ sample at 300 K, revealing a clear diamagnetic behavior under applied magnetic fields up to ±5 T.^30,31^ Beside that, at magnetic intensities lower than ±0.2 T, the material exhibited a residual ferromagnetic signal, evident after a subtraction of the diamagnetic signal as shown in the inset of Fig. 4.^30,31^ The diamagnetic behavior of Zn_0.95_Cu_0.05_O_1−*δ*_ is largely due to presence of fully filled d orbital of Zn^2+^ and Cu^+^ having electronic configuration 3d^10^4s^0^4p^0^. The weak ferromagnetism in Zn_0.95_Cu_0.05_O_1−*δ*_ can be originated due to the polarization of unpaired 2p electrons of O with the empty 4p orbital of Zn and Cu around oxygen vacancies in wurtzite lattice. Oxygen vacancy induced room temperature ferromagnetisms is observed within the percolation limit in various oxide system such as doped and un-doped ZnO, TiO_2_, CeO_2_, and SnO_2_.^31–34^ Thus the magnetization (*M*–*H*) study also confirm the oxygen vacancy formation due to incorporation of diamagnetic Cu^+^ ion on Zn^2+^ sites in ZnO lattice.

The absence of oxide ion or oxygen vacancy formation can introduce net dipole moment in Zn and Cu tetrahedral and can also destabilize the local structure/coordination and distort the local polyhedral structure of wurtzite ZnO lattice to result ferroelectric transitions. The impedance spectroscopy was carried at variable temperature in air to study the dielectric behaviour of the material in the frequency range of 100 kHz to 1 Hz. The dielectric constant was calculated using formula:3
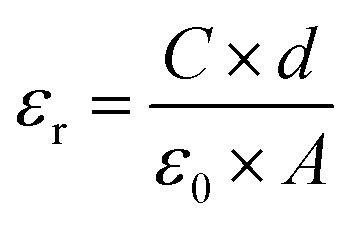
where; *ε*_r_ is the dielectric constant, *C* is capacitance, *ε*_0_ is permittivity of free space (8.85 × 10^−12^) F m^−1^, *d* is the thickness of the pellet, *A* is area of the pellet.

Capacitance (*C*) was calculated by using formula4
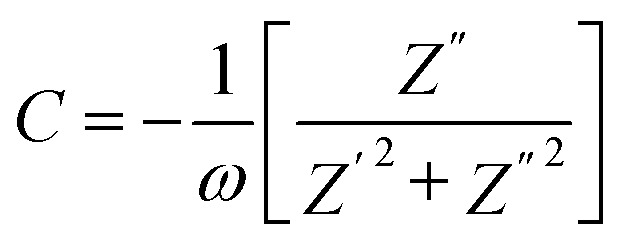
and the dielectric loss was calculated by5
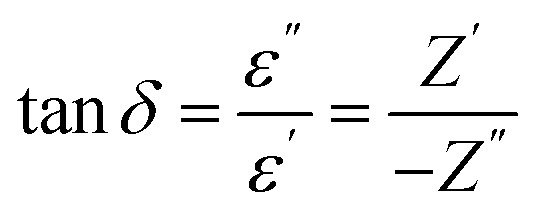


**Fig. 5:**
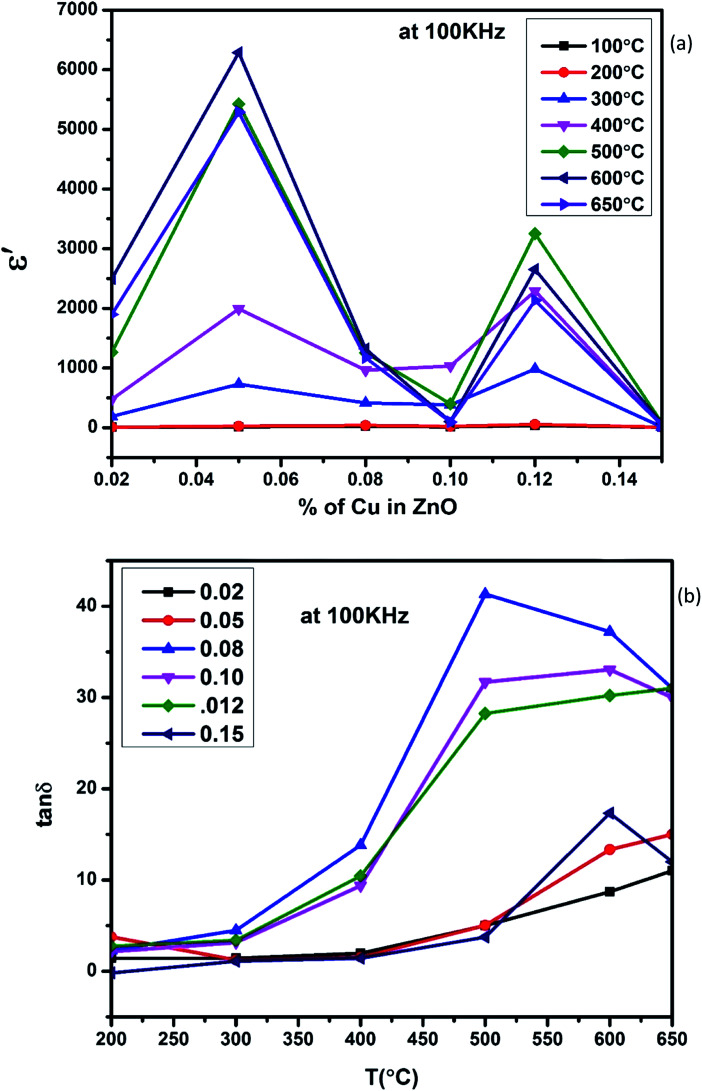
(a) Variation of dielectric constant and (b) variation of dielectric loss with respect to Cu^+^ ion substitution in ZnO lattice at 100 kHz with increase in temperature.

**Fig. 6:**
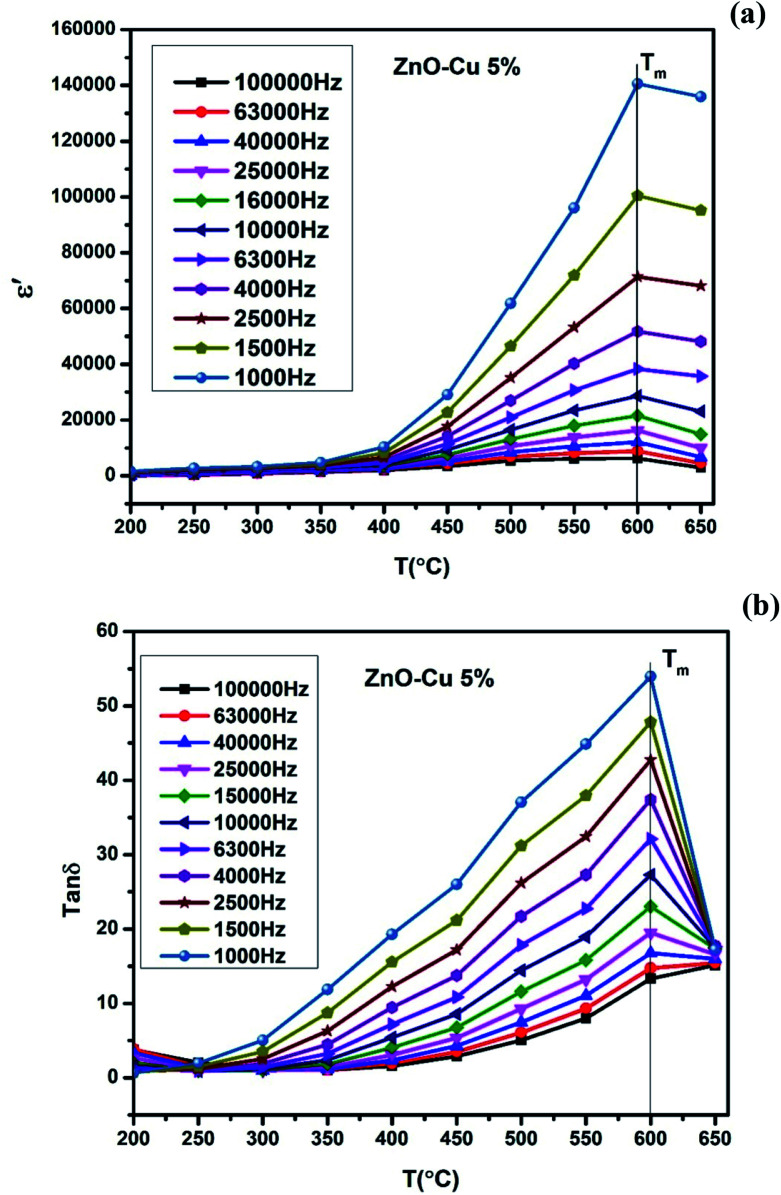
(a) Variation of dielectric constant and (b) variation of dielectric loss of Zn_0.95_Cu_0.05_O_1−*δ*_ pellets at various frequencies with increase in temperature.

**Fig. 7:**
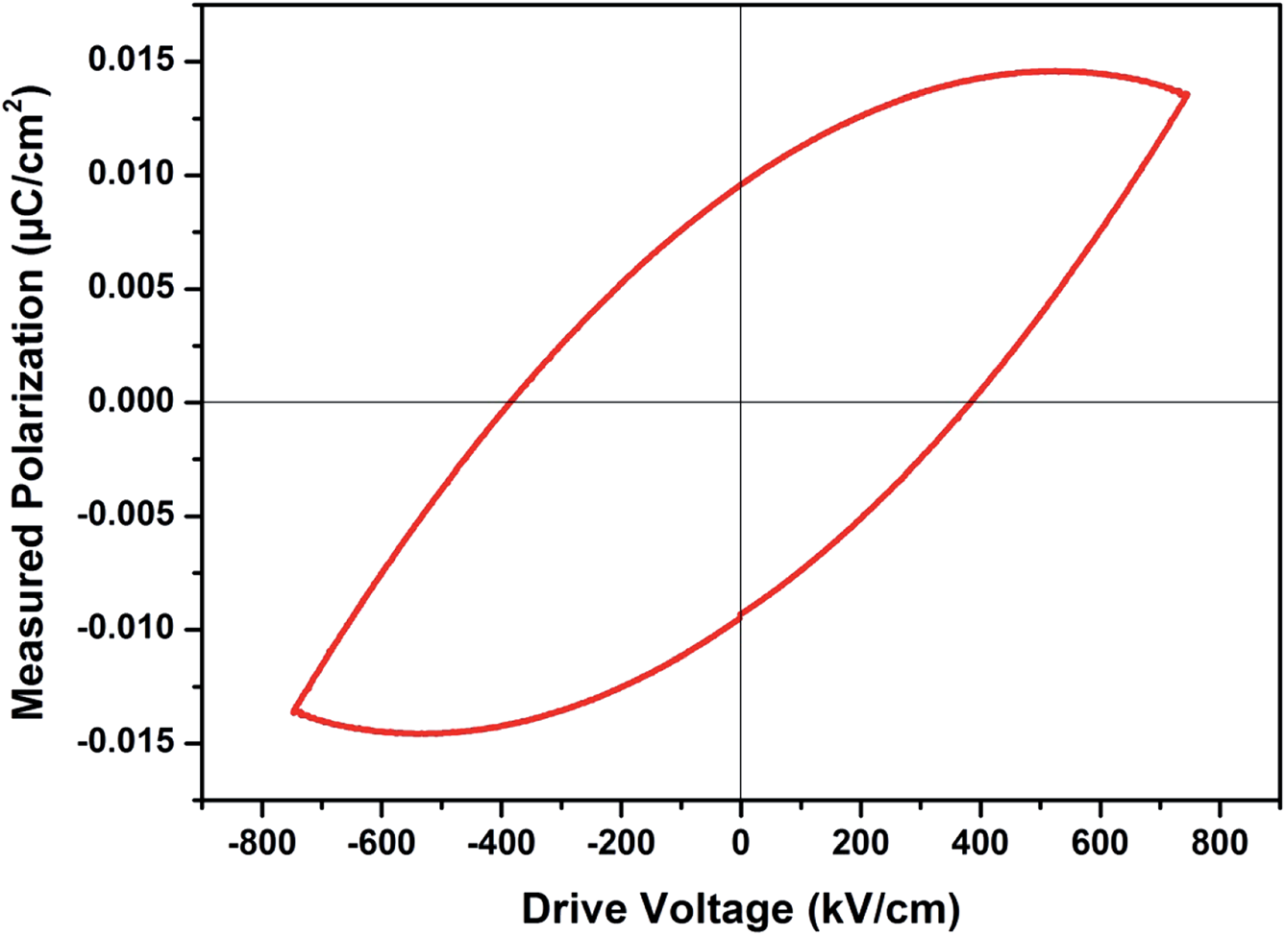
Plot of polarization (*P*) *vs.* electric field (*E*) for Zn_0.95_Cu_0.05_O_1−*δ*_ pellets at room temperature.

Fig. 5(a), show the plots of real part of dielectric constant 
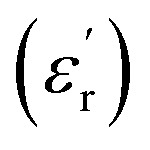
 at 100 kHz frequency (*f*) for the Zn_1−*x*_Cu_*x*_O_1−*x*/2_ (*x* = 0.02, 0.05, 0.08, 0.1, 12 and 0.15) pallets in the temperature range of 100–650 °C. In general, the 
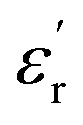
 values were increasing in the range of 100 °C to 650 °C with a maxima appearing around 600 °C. The maxima for dielectric constant 
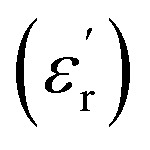
 of Zn_1−*x*_Cu_*x*_O_1−*δ*_ were appeared for *x* = 0.05 and *x* = 0.12. The dielectric constant 
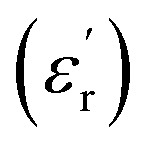
 for *x* = 0.05 was ∼6300 and for *x* = 0.12 was ∼3275 respectively at 100 kHz *f* at 600 °C. The variation in dielectric loss 
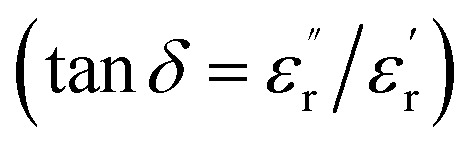
 with temperature, at selected frequency, is shown in Fig. 5(b). tan *δ* was ∼13 for *x* = 0.05 and ∼30 for *x* = 0.12 at 100 kHz frequency at 600 °C. Considering the high dielectric constant of the materials, the observed dielectric loss is quite less for Zn_1−*x*_Cu_*x*_O_1−*δ*_ samples. As can be seen from Fig. 5(a), that dielectric constant of Cu doped ZnO is varying with the doping of Cu and the highest dielectric constant (6300 at 600 °C) at 100 kHz frequency was obtained for Zn_0.95_Cu_0.05_O_1−*δ*_, the later studies were centralized around the dielectric and ferroelectric properties of Zn_0.95_Cu_0.05_O_1−*δ*_. Fig. 6(a and b) show the dielectric constant and dielectric loss for Zn_0.95_Cu_0.05_O_1−*δ*_ samples at various temperature in the range of 100–650 °C at variable frequencies. It was found that both dielectric constant and dielectric loss were increasing continuously up to 600 °C at all frequencies. However, both dielectric constant and dielectric loss were decreasing with increasing frequencies. The increase in dielectric constant 
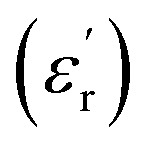
 with increasing temperatures at different frequencies for Zn_1−*x*_Cu_*x*_O_1−*δ*_ is likely due to the localized nature of hopping charge carriers in addition to interfacial polarization due to space charge. These extrinsic contributions to 
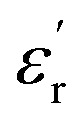
 are expected to contribute significantly only at higher frequencies. Further, it was also observed that the maximum dielectric constant and maximum dielectric loss was obtained at *T*_m_ = 600 °C between frequency range of 10 kHz to 100 kHz. This suggests that the behaviour of the material is not relaxor type as *T*_m_ is not varying with applied frequencies. Therefore *P*–*E* characteristic of the material was studied at room temperature using RADIANT precision premier II. The measured polarization hysteresis loop for Cu doped ZnO; Zn_0.95_Cu_0.05_O_1−*δ*_ is shown in Fig. 7. The observation of the clear hysteresis-loop behaviour, by a Sawyer–Tower circuit, with the external applied field confirms the existence of ferroelectricity in bulk Zn_0.95_Cu_0.05_O_1−*δ*_ pellets at room temperature. In our knowledge, this is the first report of formation of a clear *P*–*E* hysteresis-loop for any of bulk ZnO based samples. The values of remnant polarization *P*_r_ and *V*_c_ was found 9.60 × 10^−3^ (μC cm^−2^) 3.83 × 10^2^ (V cm^−1^), respectively, for Zn_0.95_Cu_0.05_O_1−*δ*_. The values of *P*_r_ and *V*_c_ are quite low, however, the formation *P*–*E* hysteresis-loop in Cu^+^ doped ZnO samples open new direction in making of binary nontoxic ferroelectrics and understanding the role oxygen vacancy and transition metal ion d orbital interaction with oxygen 2p orbital in binary oxides.

The materials Zn_0.95_Cu_0.05_O_1−*δ*_ also exhibit ferroelectricity at room temperature with remnant polarization *P*_r_ and *V*_c_ equal to 9.60 × 10^−3^ μC cm^−2^ and 3.83 × 10^2^ V cm^−1^ respectively. However the other materials of Cu and Zn systems from thin film have relative higher values for the ferroelectricity, the values of the ZnO : Cu (8 at%) was investigated. As the frequency increases, the maximum polarization decreases from ≈0.78 μC cm^−2^ at 500 Hz to ≈0.72 μC cm^−2^ at 2 kHz. The shape of the *P*–*E* loops and the remanent polarization were found to exhibit little frequency dependence in the range of 0.5 to 2 kHz.^21^ However this is the first report of ferroelectricity in the bulk samples. We believe that if our samples can be fabricated in the form of thin film, with highly aligned grain, high remnant polarization can be achieved.

## Conclusions

The quest to develop or identify ferroelectric in binary oxide materials leads us investigate oxygen vacant structure in strong incipient dielectric host such as wurtzite ZnO lattice. Cu^+^ ion substitution on Zn^2+^ sites not only resulted net polarization but also open the path for strong polarization between O 2p and fully filled d^10^ Zn/Cu 4p orbital in tetrahedral coordination. Cu^+^ ion substitution on Zn^2+^ sites in ZnO lattice is achieved by careful selection raw material CuCl and adaptation low temperature sol–gel synthesis route for the preparation of bulk material. The magnetization (*M*–*H*) study confirm the diamagnetic behavior of Zn_0.95_Cu_0.05_O_1−*δ*_ samples that is arises largely due to presence of fully filled d orbital of Zn^2+^ and Cu^+^ having electronic configuration 3d^10^4s^0^4p^0^ and also confirm the oxygen vacancy formation due to incorporation of diamagnetic Cu^+^ ion on Zn^2+^ sites in ZnO lattice. Up to 8% of Cu^+^ ions were substituted in ZnO lattice and highest dielectric constant (∼6300) was obtained at 600 °C for Zn_0.95_Cu_0.05_O_1−*δ*_ at 100 kHz frequency. The materials Zn_0.95_Cu_0.05_O_1−*δ*_ also exhibit ferroelectricity at room temperature with remnant polarization *P*_r_ and *V*_c_ equal to 9.60 × 10^−3^ μC cm^−2^ and 3.83 × 10^2^ V cm^−1^ respectively.

## Conflicts of interest

The authors declare that they have no known competing financial interests or personal relationships that could have appeared to influence the work reported in this paper.

## Supplementary Material

RA-010-D0RA00933D-s001
