# Supplementary material for: Cu(i) substituted wurtzite ZnO: a novel room temperature lead free ferroelectric and high-κ giant dielectric
Source: RSC Adv. 2020 Mar 20;10(19):11382–92. doi: 10.1039/d0ra00933d (PMC9050435; doi:10.1039/d0ra00933d)
Supplement: RA-010-D0RA00933D-s001 [file RA-010-D0RA00933D-s001.pdf]

Electronic Supplementary Material (ESI) for RSC Advances.
This journal is © The Royal Society of Chemistry 2020

Supporting Information:

Table S1. Structural Parameters of $Zn_{1-x}Cu_xO_{1-\delta}$

Compound	Lattice Parameters		$\chi 2$	R_f	R_{Bragg}	R_{wp}
	a=b	c				
$Zn_{0.98}Cu_{0.02}O_{1-\delta}$	3.249193	5.204031	1.581	6.47	7.88	14.2
$Zn_{0.95}Cu_{0.05}O_{1-\delta}$	3.251282	5.207811	2.556	7.47	6.78	15.5
$Zn_{0.92}Cu_{0.08}O_{1-\delta}$	3.251762	5.205712	2.341	6.81	7.12	15.9
$Zn_{0.9}Cu_{0.1}O_{1-\delta}$	3.251971	5.203214	2.115	7.21	6.18	16.7
$Zn_{0.88}Cu_{0.12}O_{1-\delta}$	3.252005	5.205581	2.188	6.73	7.20	17.9
$Zn_{0.85}Cu_{0.15}O_{1-\delta}$	3.251903	5.204715	3.124	7.17	6.89	17.5

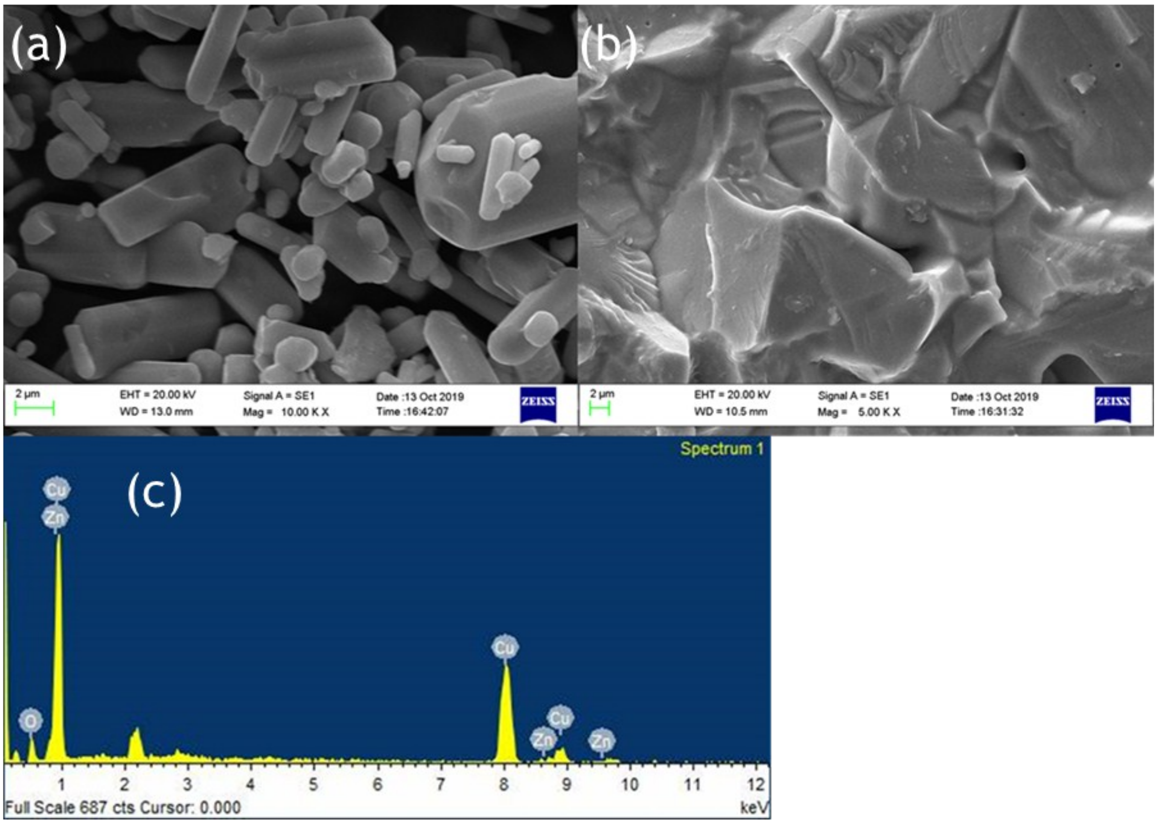

Supporting Figure S1. Scanning electron micrograph of Zn0.95$Cu_{0.05}O_{\delta}$ (a) powder and (b) Pellet, sintered at 1050°C for12h, showing clean single phase material with distinctive grains. (c) EDX analysis showing stoichiometric distribution of Zn, Cu and O elements.
